# Tumor necrosis factor-inducible gene 6 promotes liver regeneration in mice with acute liver injury

**DOI:** 10.1186/s13287-015-0019-z

**Published:** 2015-03-11

**Authors:** Sihyung Wang, Ji-Seon Lee, Jeongeun Hyun, Jieun Kim, Seung U Kim, Hyuk-Jin Cha, Youngmi Jung

**Affiliations:** Department of Intergrated Biological Science, Pusan National University, 63-2 Pusandaehak-ro, Kumjeong-gu, Pusan 609-735 Korea; Department of Biological Sciences, Pusan National University, 63-2 Pusandaehak-ro, Kumjeong-gu, Pusan 609-735 Korea; Department of Life Science, Sogang University, Seoul, 121-742 Korea; Division of Neurology, Department of Medicine, University of British Columbia, Vancouver, BC Canada

## Abstract

**Introduction:**

Tumor necrosis factor-inducible gene 6 protein (TSG-6), one of the cytokines released by human mesenchymal stem/stromal cells (hMSC), has an anti-inflammatory effect and alleviates several pathological conditions; however, the hepatoprotective potential of TSG-6 remains unclear. We investigated whether TSG-6 promoted liver regeneration in acute liver failure.

**Methods:**

The immortalized hMSC (B10) constitutively over-expressing TSG-6 or empty plasmid (NC: Negative Control) were established, and either TSG-6 or NC-conditioned medium (CM) was intraperitoneally injected into mice with acute liver damage caused by CCl_4_. Mice were sacrificed at 3 days post-CM treatment.

**Results:**

Higher expression and the immunosuppressive activity of TSG-6 were observed in CM from TSG-6-hMSC. The obvious histomorphological liver injury and increased level of liver enzymes were shown in CCl_4_-treated mice with or without NC-CM, whereas those observations were markedly ameliorated in TSG-6-CM-treated mice with CCl_4_. Ki67-positive hepatocytic cells were accumulated in the liver of the CCl_4_ + TSG-6 group. RNA analysis showed the decrease in both of inflammation markers, tnfα, il-1β, cxcl1 and cxcl2, and fibrotic markers, tgf-β1, α-sma and collagen α1, in the CCl_4_ + TSG-6 group, compared to the CCl_4_ or the CCl_4_ + NC group. Protein analysis confirmed the lower expression of TGF-β1 and α-SMA in the CCl_4_ + TSG-6 than the CCl_4_ or the CCl_4_ + NC group. Immunostaining for α-SMA also revealed the accumulation of the activated hepatic stellate cells in the livers of mice in the CCl_4_ and CCl_4_ + NC groups, but not in the livers of mice from the CCl_4_ + TSG-6 group. The cultured LX2 cells, human hepatic stellate cell line, in TSG-6-CM showed the reduced expression of fibrotic markers, tgf-β1, vimentin and collagen α1, whereas the addition of the TSG-6 antibody neutralized the inhibitory effect of TSG-6 on the activation of LX2 cells. In addition, cytoplasmic lipid drops, the marker of inactivated hepatic stellate cell, were detected in TSG-6-CM-cultured LX2 cells, only. The suppressed TSG-6 activity by TSG-6 antibody attenuated the restoration process in livers of TSG-6-CM-treated mice with CCl_4_.

**Conclusions:**

These results demonstrated that TSG-6 contributed to the liver regeneration by suppressing the activation of hepatic stellate cells in CCl_4_-treated mice, suggesting the therapeutic potential of TSG-6 for acute liver failure.

**Electronic supplementary material:**

The online version of this article (doi:10.1186/s13287-015-0019-z) contains supplementary material, which is available to authorized users.

## Introduction

Acute liver failure and chronic liver disease are life-threatening diseases for which liver transplantation is the only permanent remedy. However, the number of available organs from donors is vastly insufficient for the number of patients requiring such procedures. Even if transplant patients receive a whole liver transplantation, several post-transplant complications may arise, such as immune rejection response and death of the donor or recipient in worst-case scenarios [1]. Therefore, extensive studies are being conducted to develop new treatments for liver diseases, and stem cell based therapy has been suggested as an alternative treatment strategy for patients who suffer from various hepatic diseases [2].

Mesenchymal stem cells (MSCs) found in most adult and postnatal organs are capable of self-renewing and differentiating into several lineages of cells, including hepatocytes [3,4]. This differentiation potential of MSCs into hepatocytes provides new and promising therapeutics for patients with liver disease. These therapeutic effects of MSCs in the treatment of liver disease have been reported both in animal and clinical studies [5]. In those studies, MSCs were shown to contribute to liver regeneration by secreting tropic and immunomodulatory molecules [6,7]. However, there are still a number of technical limitations or possible undesirable side effects associated with the therapeutic application of MSCs to patients with end-stage liver diseases [8]. In particular, engrafted MSCs can differentiate into not only hepatocytes but also myofibroblasts, a main source of collagen fiber in a fibrotic liver, depending on the timeframe of differentiation and route of MSC injection [9]. Hence, further characterization of MSCs may be critical for ensuring the safety of MSC-based cell therapy. The beneficial effect of MSC transplantation is based on autologous transplantation. However, it is difficult to try MSC transplantation with patients with end-stage liver disease [9]. Although allogeneic stem cell transplantation might be more effective for these patients, it also brings several obstacles, such as immune rejection or engraftment of virus-carrying MSCs [1]. The paracrine effect, which results from biologically active soluble factors secreted from human MSCs (hMSCs), such as angiopoietin-1, interleukin-10, keratinocyte growth factor, and so on, has been shown to be therapeutically valid in both animal and clinical studies [10,11]. Since many kinds of tropic and immunomodulatory factors secreted from MSCs are also known to create a favorable micro-environment for liver regeneration [9], it is necessary to identify and characterize such biologically active soluble factors.

Tumor necrosis factor-inducible gene 6 protein (TSG-6), a 35 kDa glycoprotein [12], was identified as an inflammatory factor as its expression increased in response to inflammatory mediators [13]. However, upregulated TSG-6 during the inflammatory process has been shown to contribute to modulate the inflammatory response in adverse [13]. Recent studies demonstrate that TSG-6 is identified as an important immune modulator secreted from hMSCs and shown to be responsible for hMSCs’ therapeutic effects, such as improvement of cardiac function, peritonitis and wound healing [14]. However, these associations of TSG-6 with response in the liver are unknown.

Liver inflammation occurs in response to damage [15]. Liver injuries stimulate the repair response, such as proliferation of hepatocytes and inflammation, and a successful repair response reconstitutes a functional liver [16]. However, continued damage perpetuates injury and promotes progressive fibrogenesis [16]. Liver inflammation also accelerates fibrosis [17]. Several inflammatory factors, such as tumor necrosis factor (TNF-α) and interleukin (Il) -1β, and Il-6, secreted by inflammatory cells are involved in the recruitment of circulating macrophages into the liver and the transition of hepatic stellate cells into myofibroblasts [18]. This progressive fibrogenesis ends with death from cirrhosis and/or liver cancer [19]. Thus, the modulation of liver inflammation in this setting is a key target for reducing fibrosis. For this reason, we hypothesized that TSG-6 could influence liver regeneration by reducing inflammation and fibrosis because TSG-6 exerts an anti-inflammatory effect. To prove our hypothesis, we made immortalized hMSCs stably expressing TSG-6. Due to constant secretion of TSG-6 from hMSCs in the conditioned medium (CM), TSG-6-rich CM could be readily applied to mice with acute liver injury caused by CCl_4_. Our results showed that TSG-6 promoted liver regeneration by decreasing fibrosis.

## Methods

### Generation of hMSCs stably expressing TSG-6

We sub-cloned P/I-TSG-6 (kindly provided by Dr. Tae-Hee Lee in Yonsei Univ.) into a CSII-EF-MCS vector. hMSCs stably expressing TSG-6, were generated as previously reported [20]. In detail, each viral plasmid (3 μg) (CSII-EF-MCS and CSII-EF-TSG-6) was transfected into 293FT cells with pLP1, pLP2, pLP/VSVG, using lipofectamine 2000 (cat. #11668-027, Life Technologies, Inc. Grand Island, NY, USA). After 48 hours, the culture media containing the lenti-viruses were collected from the transfected 293FT cells. Virus medium was filtered (0.45 μm filter, EMD Millipore, Billerica, MA, USA) and then treated to hMSCs for an additional 24 hours in the presence of 4 μg/ml of polybrene (Sigma–Aldrich, St. Louis, MO, USA). B10-hMSCs were maintained as described previously [21].

### Preparation of conditioned media

For preparation of CM, cells were seeded at more than 90% confluence. The next day, cells were washed with PBS twice, changed to 0.2% fetal bovine serum (FBS)-containing media and incubated for another three days. CM were further concentrated with YM-10 (Millipore, Cat. No. 4205).

### Immune suppression assay

Splenocytes, 1.5 × 10^6^ per well (24-well) (freshly isolated from C57BL6 female mice as described previously [22]), were cultured with or without CM supplement for three days. To stimulate T cells, 1 μg/ml of anti-CD3ε (2C11) (BD Pharmingen, San Jose, CA, USA) was added. Cells were fluorescence labeled with carboxyfluorescein succinimidyl ester (CFSE) and then proliferation after activation was determined by flow cytometry (BD, FACS Calibur™).

### Animal studies

**Figure 1:**
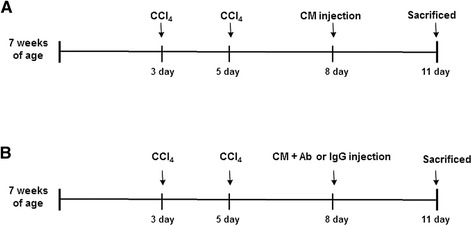
**Design of mouse experimental model. (A)** After being i.p. injected with CCl_4_ or corn oil (CTRL) twice for one week, mice were treated with NC-CM or TSG-6-CM, one time at eight days. These mice were sacrificed at 11 days. **(B)** CCl_4_-treated mice were injected with the neutralized TSG-6-CM by TSG-6 antibody or the TSG-6-CM + IgG antibody. These mice were also sacrificed at 11 days. CM, conditioned medium; CTRL, control; IgG, immunoglobulin G; i.p., intraperitoneally; NC, negative control; TSG-6, tumor necrosis factor-inducible gene 6 protein.

Six-week old male C57BL6 mice were purchased from Hyochang (Dae-gu, Korea), fed with a normal diet, watered, and housed with a 12 hour light-dark cycle. *In vivo* experiments were performed as previously described. Mice were seven weeks of age and weighed an average of 21 g at the start of the experiments. To induce acute liver injury, mice received CCl_4_ (1.0 mg/kg body weight) (n = 15) intraperitoneally twice for one week as previously described [23]. As controls, animals received the same volume of corn oil vehicle (CTRL) (n = 4) intraperitoneally. Those mice were then injected i.p. with 0.5 ml CM from TSG-6 overexpressing MSCs (TSG-6) (n = 6), or empty plasmid-carrying MSCs (NC; negative control) (n = 5) at day 8, and sacrificed at day 3 following the medium treatment (Figure 1A). To examine the specificity of TSG-6 for liver, TSG-6-CM was incubated with TSG-6 antibody (10 μg/ml) (Santa Cruz Biotechnology, Santa Cruz, CA, USA) or control immunoglobulin G (IgG) (10 μg/ml) (Sigma-Aldrich) for one hour at room temperature, and 0.5 ml of these CMs was injected into CCl_4_-treated mice (TSG-6-CM + TSG-6 antibody group: n = 5/ TSG-6-CM + IgG group: n = 5) (Figure 1B).

Animal care and surgical procedures were approved by the Pusan National University Institutional Animal Care and Use Committee and carried out in accordance with the provisions of the National Institutes of Health Guide for the Care and Use of Laboratory Animals.

### Measurement of AST/ALT

Serum aspartate aminotransferase (AST) and alanine aminotransferase (ALT) were measured using Chemi Lab GOT/GPT (IVD Lab Co., Korea) according to the manufacturer’s instructions.

### Liver histology and immunohistochemistry

Liver specimens were fixed in 10% neutral buffered formalin, embedded in paraffin and cut into 4 μm sections. Specimens were dewaxed, hydrated, and stained in the usual manner with standard hematoxylin and eosin (H & E) to examine morphology. For immunohistochemistry (IHC), sections were incubated for 10 minutes in 3% hydrogen peroxide to block endogenous peroxidase. Antigen retrieval was performed by heating in 10 mM sodium citrate buffer (pH 6.0) or incubating with pepsin for 10 minutes. After washing with TBS, sections were treated with Dako protein block (X9090; Dako Envision, Carpinteria, CA) for 30 minutes and incubated with primary antibody Ki67 (NCL-Ki67, Novocastra, Leica Microsystems, Newcastle, Upon Tyne, UK), pancytokeratin (PanCK) (Z0622; Dako), Sox9 (AB5535; Millipore), or α-Sma (ab5694; Abcam, Cambridge, Massachusetts, USA), at 4°C overnight. Other sections were also incubated at 4°C overnight in non-immune sera to demonstrate staining specificity. Polymer horseradish peroxidase (HRP) anti-rabbit (K 4003; Dako) or anti-mouse (K 4001; Dako) was used as secondary antinody. 3,3′-Diaminobenzidine (DAB) was employed in the detection procedure.

### Cell counting

To quantify the number of Ki67-positive cells, 10 central vein (CV) areas were randomly selected per section at × 40 magnification for each mouse. CV chosen for analysis ranged from 90 to 150 μm. The Ki67-positive cells were quantified by counting the total number of Ki67-positive cells per field.

### Quantitative real-time PCR

Total RNA which had been stored at -80°C was extracted with TRIZOL™ (Ambion® by Life Technologies). After assuring RNA quality and concentration, gene expression was evaluated by QRT-PCR analysis. mRNAs were quantified by real-time RT-PCR according to the manufacturer’s specifications (Eppendorf, Mastercycler Real-TIme PCR, Effendorf Korea Ltd., Seoul, Korea). The sequences of primers for mice are listed in Table 1. Samples were analyzed in duplicate according to the ΔΔCt method. All PCR products were directly sequenced for genetic confirmation in Macrogen Inc (Korea).

**Table 1:** **Sequences of primers used for QRT-PCR**

**Mouse**		
**Gene**	**Forward sequence**	**Reverse sequence**
g6pc	TCCTCCTCAGCCTATGTCTGCATTC	GAGAGAAGAATCCTGGGTCTCCTTG
tgf-β1	TTGCCCTCTACAACCAACACAA	GGCTTGCGACCCACGTAGTA
α-sma	AAACAGGAATACGACGAAG	CAGGAATGATTTCCAAAGGA
collagen *α1*	GAGCGGAGAGTACTGGATCG	GCTTCTTTTCCTTGGGGTTC
tnf-α	TCGTAGCAAACCACCAAGTG	ATATAGCAAATCGGCTGACG
il-1β	ACTCCTTAGTCCTCGGCCA	TGGTTTCTTGTGACCCTGAGC
cxcl1	CCCAAACCGAAGTCATAGCC	TCAGAAGCCAGCGTTCACC
cxcl2	GCCCAGACAGAAGTCATAGCC	TTCTCTTTGGTTCTTCCGTTGA
9 s	GACTCCGGAACAAACGTGAGG	CTTCATCTTGCCCTCGTCCA
**Rat**		
**Gene**	**Forward Sequence**	**Reverse Sequence**
tsg-6	AGTGATGCGTCCGTCACAGCC	AGATGGCTAAACCGTCCAGCTAAGA
9 s	GACTCCGGAACAAACGTGAGGT	CTTCATCTTGCCCTCGTCCA
**Human**		
**Gene**	**Forward sequence**	**Reverse sequence**
tgf-β1	TTGACTGAGTTGCGATAATGTT	GGGAAATTGCTCGACGAT
vimentin	CGAAAACACCCTGCAATCTT	GTGAGGTCAGGCTTGGAAAC
collagen *α1*	CAGATCACGTCATCGCACAA	TGTGAGGCCACGCATGAG
9 s	GACTCCGGAACAAACGTGAGGT	CTTCATCTTGCCCTCGTCCA

### Western blot assay

Total protein was extracted from freeze-clamped liver tissue samples that had been stored at -80°C. Whole tissues were homogenized in RIPA (78510; Thermo, Rockford, IL) supplemented with protease inhibitors (Complete Mini 11 836 153 001; Roche, Indianapolis, IN). Equal amounts of total protein (120 μg) were fractionated by polyacrylamide gel electrophoresis and transferred to polyvinylidene difluoride (PVDF) membranes. Primary antibodies against TGF-β (3711S; Cell Signaling) and α-Sma (A5228-200UL; Sigma-Aldrich) were used in this experiment. Membranes were developed by chemiluminescence (ATTO Corporation, Tokyo, Japan). The blots that were obtained from three independent experiments were scanned and a region of interest (ROI) around the band of interest was defined. Band intensities were calculated by using the CS analyzer 2.0 program (ATTO Corporation).

### Cell experiments

The human hepatic stellate cell line LX2, a well-characterized cell line derived from human hepatic stellate cells [24], B10-NC and B10-TSG-6 were cultured at a density of 2 × 10^6^ in Minimum Essential Medium alpha (MEM α, Gibco, Life Technologies) supplemented with 10% FBS (Gibco, Life Technologies, Grand Island, NY) and 1X antibiotics at 37°C in a humidified atmosphere containing 5% CO2. LX2 was a generous gift from Won-il Jung (Korea Advanced Institute of Science and Technology). When B10-NC or B10-TSG-6 cells were 70% to 80% confluent, CM of these cells were collected to treat LX2. For biochemical analysis of gene expression changes, LX2 at 70% to 80% confluence were starved in medium containing no FBS for six hours. Activation of LX2 was verified by examining the expression of pro-fibrotic signaling genes at 24 and 48 hours after addition of FBS. Based on the gene expression data, we considered LX2 as fully activated cells at 48 hours (data not shown). Fully activated LX2 was cultured in LX2 cell medium, B10-NC-CM or TSG-6-CM. In addition, those cells cultured in TSG-6-CM were treated with TSG-6 antibody (10 μg/ml) or control IgG (10 μg/ml). These experiments were repeated three times [25].

### Statistical analysis

Results are expressed as the mean ± standard deviation (SD). Statistical differences were determined by Student’s t-test or one-way analysis of variance (ANOVA) using the SPSS statistics 20, followed by Scheffe’ *post hoc* test. *P*-values <0.05 were considered to be statistically significant.

## Results

### Generation of TSG-6-overrexpressing MSCs

**Figure 2:**
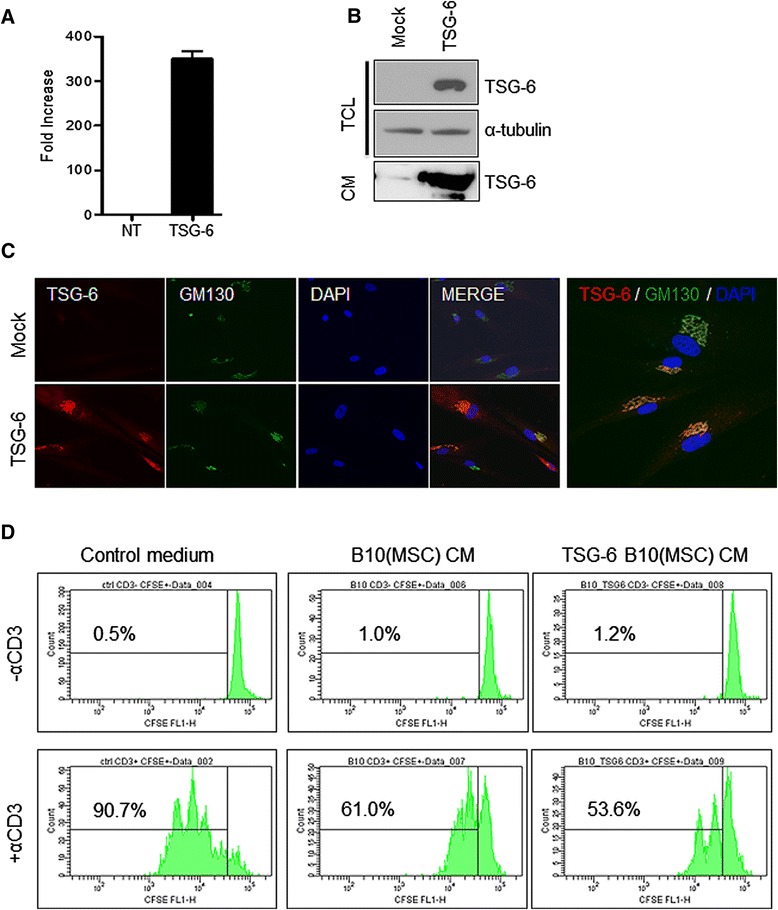
**Production of MSC overexpressing TSG-6. (A)** The mRNA level of TSG-6 in CSII-EF-MCS- and CSII-EF-TSG-6-expressing hMSC cells was determined via real-time PCR. **(B)** TSG-6 stable transfection to hMSCs was confirmed by immunoblotting for Wip1 (top panel). Equal protein loading was verified by α-tubulin immunoblotting (middle panel). Secretion of TSG-6 in hMSCs was confirmed in the conditioned media by immunoblotting for TSG-6 (bottom panel). **(C)** TSG-6 expression in TSG-6-hMSC was validated by immunostaining with anti-TSG-6 antibody (left panel, TSG-6). GM130 was counterstained as a Golgi complex marker (middle panel, GM130). DAPI was used for nuclear counterstaining (right panel, DAPI). **(D)** CD4^+^ T cells from mouse splenocytes were cultured in the presence of CD3 antibody (1 μg/ml of α-CD3). T cells proliferation was determined by FACS analysis after CFSE staining. CM either from hMSCs (B10) or TSG-6-hMSCs were incubated for three days in the presence of CD3 antibody. Culture medium for hMSCs was used as the control medium. CFSE, carboxyfluorescein succinimidyl ester; CM, conditioned medium; DAPI, 4′,6-diamidino-2-phenylindole; FACS, fluorescence activated cell sorting; hMSCs, human mesenchymal stem cells; MSCs, mesenchymal stem cells; TSG-6, tumor necrosis factor-inducible gene 6 protein.

To investigate the effect of TSG-6 in liver injury, we generate hMSCs stably expressing TSG-6 based on immortalized fetal bone marrow hMSCs (B10) [21], using a lentivirus delivery system. The stable expression of TSG-6 in hMSCs was confirmed by a higher expression level of TSG-6 mRNA (Figure 2A) and protein (Figure 2B) in TSG-6-hMSCs. More importantly, a detectable level of TSG-6 protein was also found in the CM from TSG-6-hMSCs (Figure 2B, lower panel). Given that TSG-6, a glycoprotein is processed in the Golgi complex, a distinct signal from the Golgi complex (determined by GM130, a typical Golgi marker) in TSG-6-hMSCs was clearly observed (Figure 2C). To examine the immune-modulatory activity of TSG-6 secreted from TSG-6-hMSCs, splenocytes freshly isolated from the mouse spleen were treated with CM from hMSCs (B10) or TSG-6-hMSCs (TSG-6 B10). While activated CD4^+^ T cells by CD3 antibody underwent active proliferation, proliferation of CD4^+^ T cells was markedly reduced following treatment with CM from either hMSC or TSG-6-hMSCs, compared to control medium even after CD3 stimulation. Of interest, CM from TSG-6-hMSCs appeared to be more effective in suppressing T cell proliferation (Figure 2D).

### TSG-6 attenuates liver injury

**Figure 3:**
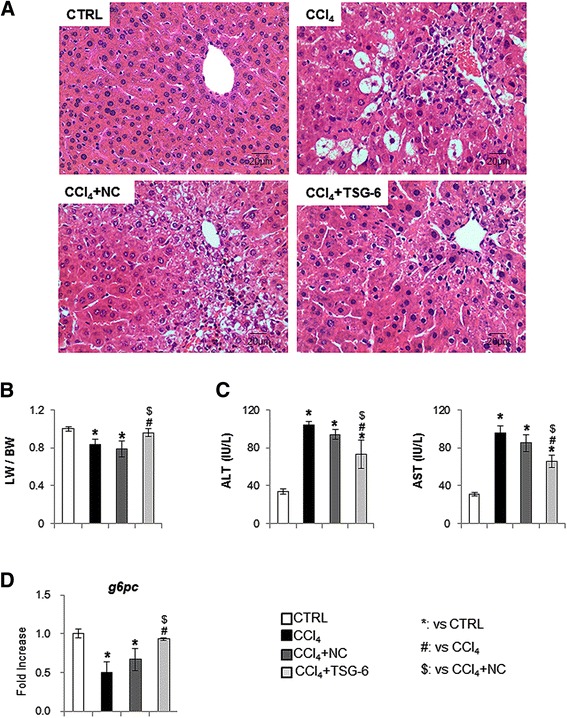
**Effects of TSG-6 on liver histomorphology and function in CCl**_**4**_ **-treated mice. (A)** H & E staining shows the extensive cellular damage and infiltration of inflammatory cells in CCl_4_-treated mice with or without NC (negative control: mock transfected cell)-conditioned medium (CM). Those cellular injuries were reduced in CCl_4_ mice treated with TSG-6-CM (CCl_4_ + TSG-6). The representative images are shown at × 40 (CTRL: corn-oil-treated control mice/ CCl_4_:CCl_4_-treated mice/ CCl_4_ + NC: CCl_4_-treated mice with NC-CM). **(B)** Relative liver weight / body weight of mice. **(C)** The values of AST and ALT are graphed. **(D)** QRT-PCR analysis for G6pc of liver mRNA from normal (CTRL), CCl_4_, CCl_4_ with NC-CM (NC) or TSG-6-CM (TSG-6) (n ≥4 mice / group) (ANOVA, *P <0.05 versus CTRL, #*P* <0.05 versus CCl_4,_ $*P* <0.05 versus CCl_4_ + NC-CM). ALT, alanine aminotransferase; ANOVA, analysis of variance; AST, aspartate aminotransferase; G6pc, glucose-6-phosphatase; TSG-6, tumor necrosis factor-inducible gene 6 protein.

To examine whether TSG-6 influenced liver regeneration, mice were treated with CCl_4_ to induce acute liver injury and i.p. injected with TSG-6-rich CM (TSG-6-CM) (CCl_4_ + TSG-6-CM: TSG-6 group) or TSG-6-poor CM (NC-CM) (CCl_4_ + NC-CM: NC group). Liver sections from those mice were examined for the effect of TSG-6 using H & E staining. CCl_4_-treated mice showed severe cytoplasmic vacuolation, microvesicular fatty changes, loss of cellular boundaries, infiltration of inflammatory cells around the central vein and in the portal areas, congestion in the sinusoids, and necrosis of the liver cells. Interestingly, those abnormal morphological changes were remarkably ameliorated and restored to almost normal morphology in the TSG-6 group, compared to the NC and CCl_4_ groups (Figure 3A). The ratio of liver weight versus body weight (LW/BW) declined in mice in the CCl_4_ and NC groups (CCl_4_: 0.836 ± 0.056, NC: 0.788 ± 0.086, compared to control, *P <0.05), whereas there was no significant change of LW/BW between the control and TSG-6 groups (Figure 3B). In addition, CCl_4_-treated mice with or without NC-CM had elevated serum AST and ALT, whereas CCl_4_-treated mice with TSG-6-CM had alleviated AST (control-31.32 ± 1.74, CCl_4_-95.72 ± 7.23, NC-85.09 ± 8.59 and TSG-6-5.84 ± 6.28) and ALT (control-33.95 ± 3.09, CCl_4_-104.52 ± 3.09, NC-94.33 ± 4.79, and TSG-6-73.25 ± 15.03) (Figure 3C). Furthermore, the RNA level of glucose-6-phosphatase (G6pc), which is known to be an essential enzyme for glycolysis, was similar between the control and TSG-6 groups (Figure 3D).

**Figure 4:**
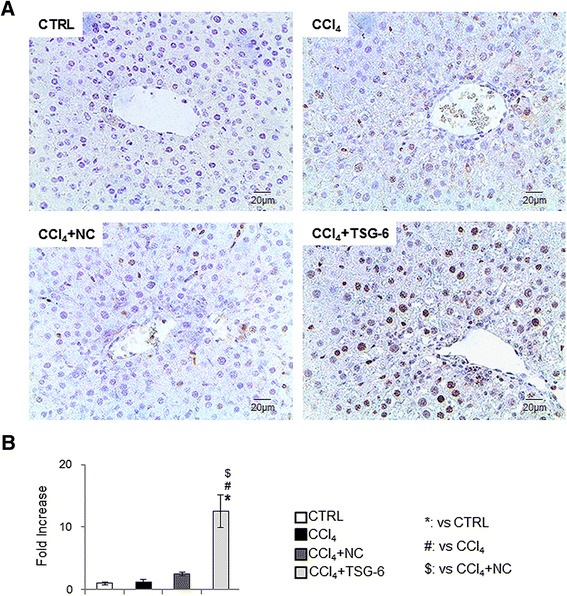
**Expansion of Ki67-positive hepatocytic cells in liver of mice with CCl**_**4**_ **+ TSG-6 treatment. (A)** IHC for Ki67 in liver sections from representative control, CCl_4_, CCl_4_ + NC, and CCl_4_ + TSG-6 mice (×40). **(B)** Ki67-positive hepatocytes were counted and graphed (ANOVA, *P <0.05 versus CTRL, #*P* <0.05 versus CCl_4,_ $*P* <0.05 versus CCl_4_ + NC-CM). ANOVA, analysis of variance; CM, conditioned medium; CTRL, control; IHC, immunohistochemistry; NC, negative control; TSG-6, tumor necrosis factor-inducible gene 6 protein.

**Figure 5:**
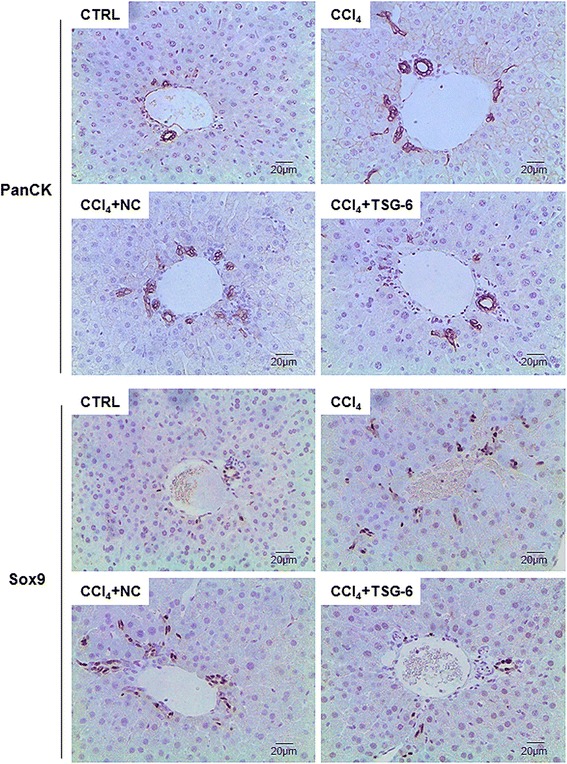
**TSG-6 decreases the proliferation of hepatic progenitors in the liver damaged with CCl**_**4**_ **.** IHC for PanCK and Sox9 in liver sections from representative control, CCl_4_, CCl_4_ + NC, and CCl_4_ + TSG-6 mice (×40). IHC, immunohistochemistry; NC, negative control; PanCK, pancytokeratin; TSG-6, tumor necrosis factor-inducible gene 6 protein.

To determine if these changes in the liver repair process were associated with the hepatocyte proliferation, liver sections from CM-treated mice with CCl_4_ were stained for Ki67, a marker of S phase [26]. The injured livers of mice with or without NC-CM mainly had Ki67-positive hepatic stellate-looking cells, whereas livers of mice with TSG-6-CM largely contained Ki67-positive hepatocytic cells (Figure 4). Because liver damage leads to expansion of liver progenitors [26,27], it was further investigated whether reduced liver injuries influenced the proliferation of liver progenitors. As assessed by IHC for PanCK and Sox9, two different markers of liver progenitors [28-30], both the CCl_4_ and NC groups exhibited an expansion of hepatic progenitors compared to the control and TSG-6 groups (Figure 5). Therefore, these results suggested that TSG-6 promoted the repair process into the normal restoration of the liver by contributing to hepatocyte proliferation.

### Decreased hepatic fibrosis in TSG-6 treated mice

**Figure 6:**
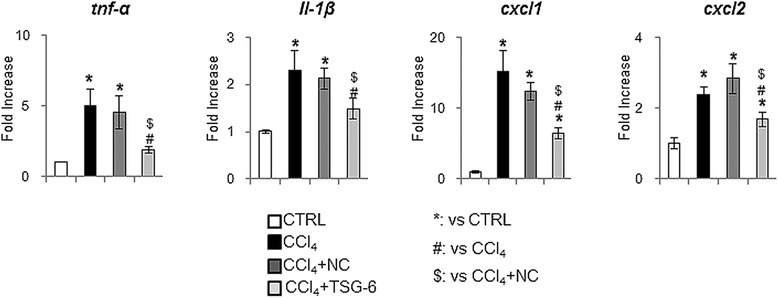
**TSG-6 decreases the inflammation in the liver damaged with CCl**_**4**_ **.** QRT-PCR analysis of mouse liver for tnfα, il-1β, cxcl1 and cxcl2. Mean ± SD results are graphed (n ≥4 mice/group) (ANOVA, *P <0.05 versus CTRL, # *P* <0.05 versus CCl_4,_ $*P* <0.05 versus CCl_4_ + NC-CM). ANOVA, analysis of variance; CM, conditioned medium. CTRL, control; NC, negative control; SD, standard deviation; TSG-6, tumor necrosis factor-inducible gene 6 protein.

**Figure 7:**
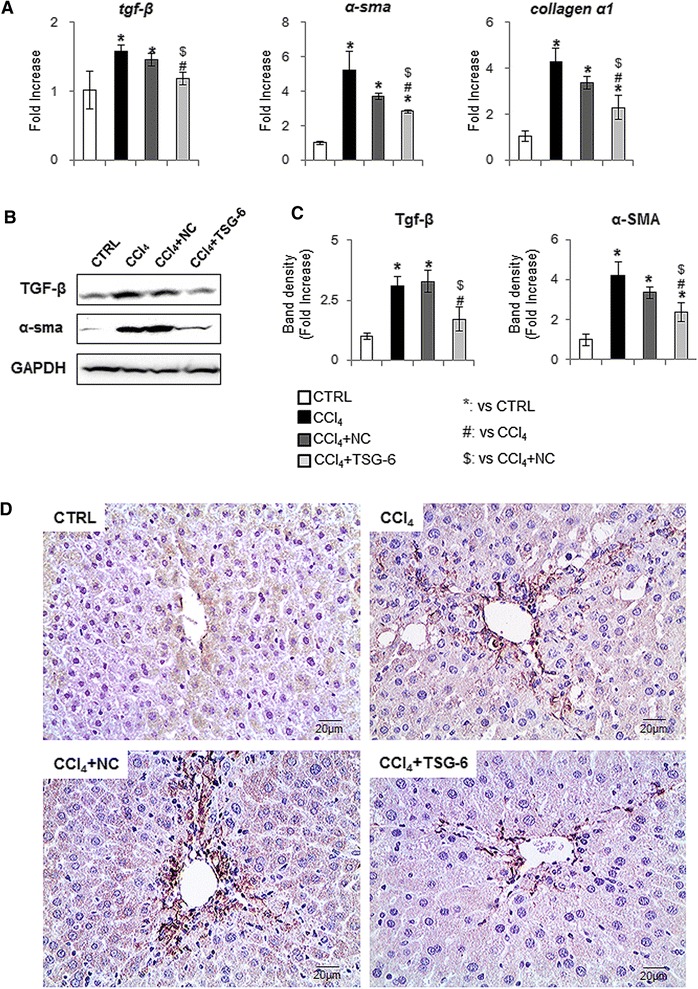
**Reduced fibrosis in the TSG-6 treated liver. (A)** QRT-PCR analysis of liver mRNA for tgf-β, α–sma and collagen-α1 (n ≥4 mice/group). Mean ± SD results are graphed. **(B)** and **(C)**. Western blot analysis of TGF-β (25 kDa: processed form) (inducer of fibrosis) and α-SMA (fibrogenic marker) (GAPDH was used as an internal control) (n ≥4 mice/group). Data shown represent one of three experiments with similar results (B: Immunoblot/ C: Band density of TGF-β and α-SMA). Data represent the mean ± SD of three independent experiments. **(D)** IHC for α-SMA in liver sections from representative control, CCl_4_, CCl_4_ + NC, and CCl_4_ + TSG-6 mice (×40) (ANOVA, *P <0.05 versus CTRL, #*P* <0.05 versus CCl_4,_ $*P* <0.05 versus CCl_4_ + NC-CM). ANOVA, analysis of variance; CM, conditioned medium; CTRL, control; IHC, immunohistochemistry; NC, negative control; SD, standard deviation; TSG-6, tumor necrosis factor-inducible gene 6 protein.

Because CCl_4_ is a well-known chemical that induces hepatocyte injury and inflammation, promoting collagen deposition, we examined whether TSG-6 might exert an anti-inflammatory effect, thereby decreasing fibrogenesis in this animal model. The expression of inflammation markers, such as tnfα, il-1β, cxcl1 and cxcl2, was significantly higher in the CCl_4_ and NC groups than in the TSG-6 group (Figure 6). The RNA expression of the fibrotic markers, tgf-β, α-sma and collagen α1, was greatly upregulated in the CCl_4_ and CCl_4_ + NC-CM-treated livers, whereas those genes were downregulated in injured livers treated with TSG-6-CM (Figure 7A). In line with mRNA expression, the protein level of TGF-β and α-SMA decreased in the TSG-6 treated group, showing the baseline TGF-β and α-SMA expression in healthy livers (Figure 7B, C). In addition, IHC staining for α-SMA clearly showed that the accumulation of activated hepatic stellate cells in livers of the CCl_4_ and NC groups was reduced in the livers of the TSG-6 group (Figure 7D). Therefore, these results demonstrated that the TSG-6 attenuated both inflammation and the expansion of fibrous matrix in the injured liver.

### TSG-6 induced inactivation of hepatic stellate cells

**Figure 8:**
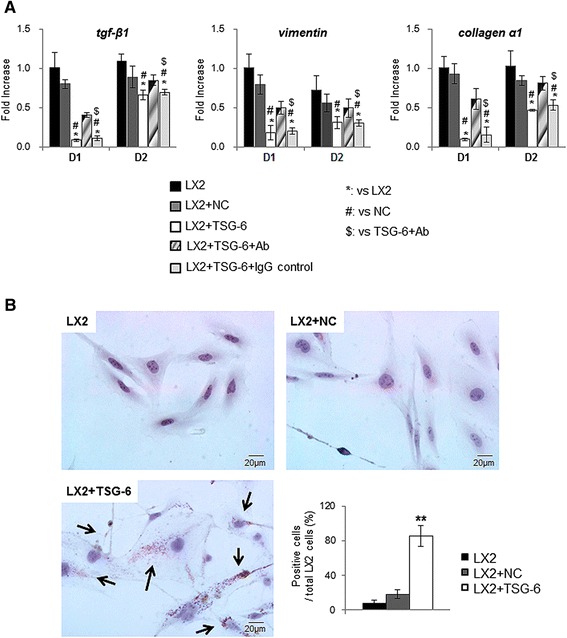
**TSG-6 blocks activation of hepatic stellate cells. (A)** QRT-PCR analysis for tgf-β, vimentin and collagen-α1 in LX2 cells which were cultured in LX2 cell medium (LX2), NC-CM (LX2 + NC), TSG-6-CM with (LX2 + TSG-6 + Ab) or without TSG-6 antibody (LX2 + TSG-6). IgG was used as a negative control for TSG-6 antibody (LX2 + TSG-6 + IgG). Mean ± SD results are graphed. Data represent the mean ± SD of three independent experiments (ANOVA, *P <0.05 versus LX2, #*P* <0.05 versus NC, $*P* <0.05 versus TSG-6 + Ab). **(B)** Oil Red O staining in LX2 cells cultured in LX2, NC-CM, or TSG-6-CM (original magnification × 40). ANOVA, analysis of variance; CM conditioned medium; IgG, immunoglobulin G; NC, negative control; SD, standard deviation; TSG-6, tumor necrosis factor-inducible gene 6 protein.

Fibrosis is the excessive accumulation of collagen and other extracellular matrix components, and the accumulated fibrotic tissues disarrange the liver constitution. A change in the hepatic structure induces hepatic dysfunction, leading to the death of the patient. Several types of cells, such as bone marrow-derived cells, circulating fibrocytes, and portal fibroblasts, are known to contribute to hepatic fibrosis by transitioning to myofibroblasts. Particularly, activated hepatic stellate cells are primary sources of myofibroblastic cells promoting fibrogenesis. Hence, we investigated whether TSG-6 was involved in the activation of hepatic stellate cells because our data showed regressed fibrosis in the TSG-6 treated group. LX2 cells cultured in TSG-6-CM showed decreased expression of activated markers of hepatic stellate cells, such as tgf-β, vimentin and collagen α1, whereas cells cultured in NC-CM or LX2 cell medium had greatly increased expression of those markers. In addition, neutralization of TSG-6 by the TSG-6 antibody effectively induced the expression of those markers in LX2 cells (Figure 8A). Oil Red O staining showed cytoplasmic lipid droplets, the morphologic hallmark of inactivated hepatic stellate cells, in the LX2 cells cultured in TSG-6-CM, whereas those lipid droplets were not seen in cells cultured in NC-CM or LX2 cell medium (Figure 8B).

**Figure 9:**
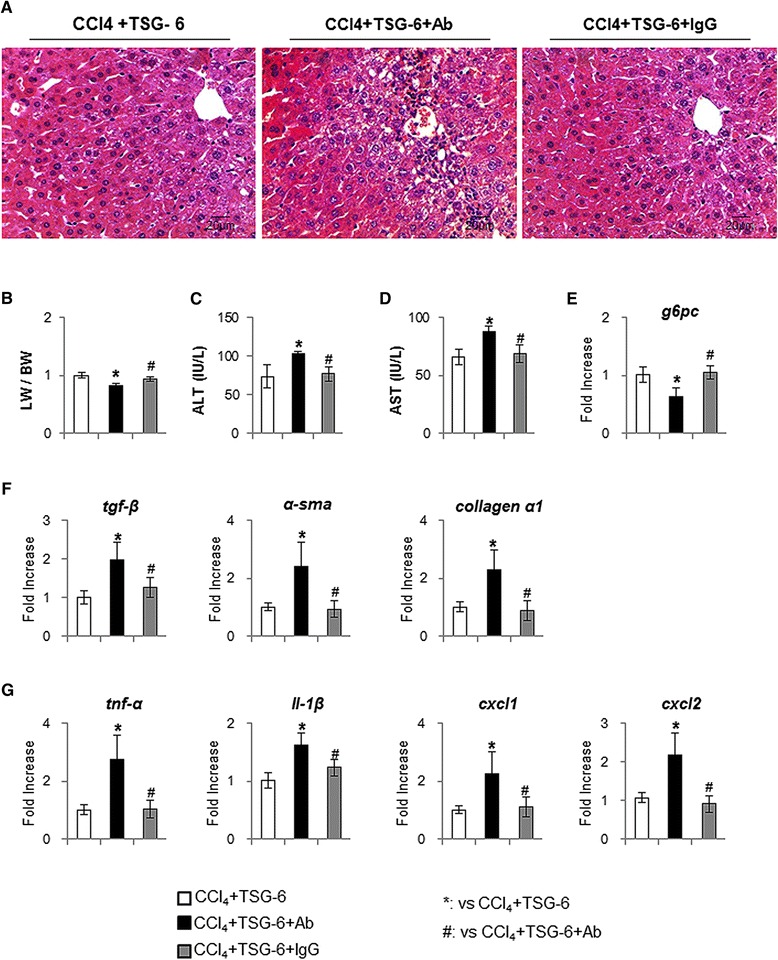
**Neutralization of TSG-6 by TSG-6 antibody attenuates the restoration effect of TSG-6 in liver of CCl**_**4**_ **+ TSG-6-treated mice. (A)** H & E staining shows the histomorphological changes in CCl_4_ + TSG-6-treated mice with (CCl_4_ + TSG-6 + TSG-6 antibody) (CCl_4_ + TSG-6 + IgG) without TSG-6 antibody (CCl_4_ + TSG-6) or IgG. The representative images were shown at × 40. **(B)** Relative liver weight/body weight of mice. **(C)**, **(D)** The values of AST and ALT are graphed. **(E**, **F**, **G)** QRT-PCR analysis for G6pc (E), the fibrotic markers (F), tgf-β, α–sma and collagen-α1, the inflammation markers (G), tnfα, il-1β, cxcl1 and cxcl2, of liver mRNA from the treated mice (n ≥4 mice / group). Mean ± SD results are graphed (ANOVA, *P <0.05 versus TSG-6, #*P* <0.05 versus TSG-6 + Ab). ALT, alanine aminotransferase; ANOVA, analysis of variance; AST, aspartate aminogransferase; G6pc, glucose-6-phosphatase; IgG, immunoglobulin G; SD, standard deviation; TSG-6, tumor necrosis factor-inducible gene 6 protein.

To assess whether TSG-6 directly influenced liver restoration in mice with CCl_4_ damage, TSG-6-CM neutralized with TSG-6 antibody was injected in CCl_4_-treated mice (TSG-6 + Ab group) (Figure 1B). The livers of the TSG-6 + Ab group had severe hepatic injuries, whereas the livers from the TSG-6 or TSG-6 + IgG groups showed almost normal morphology without distinct morphological differences between the two groups, as examined by H & E staining (Figure 9A). The ratio of liver weight/body weight (LW/BW) decreased and serum AST and ALT increased in mice in the TSG-6 + Ab group, compared with mice in the TSG-6 or TSG-6 + IgG group (Figure 9B, C, D). The RNA expression of g6pc was also down-regulated in the TSG-6 + Ab group (Figure 9E). Furthermore, the neutralization of TSG-6 by TSG-6 antibody led to an increase in the fibrotic markers, tgf-β, α-sma and collagen α1, as assessed by quantitative real-time PCR (Figure 9F). In addition, the expression of inflammation markers, tnf-α, il-1β, cxcl1 and cxcl2, was higher in the TSG-6 neutralized group than in the TSG-6 or TSG-6 + IgG groups. Taken together, these data suggested that TSG-6 induced the inactivation of hepatic stellate cells, contributing to the ameliorated fibrosis in mice.

## Discussion

Acute liver failure shows a higher inflammatory response to liver damage, which impairs the parenchymal function that extends into systemic organ failure, eventually leading to death [16,31]. Thus, researchers must develop new treatments for acute liver disease. The potential for MSCs to differentiate into hepatocytes and their immunomodulatory capabilities make MSCs an attractive choice in the therapy of acute or chronic liver diseases [9]. Several clinical trials are currently being performed to investigate the therapeutic potential of MSCs in the treatment of chronic liver diseases, such as Hepatitis B or C virus-infected liver or alcoholic liver with cirrhosis [32-34]. Previous studies have shown that transplanted MSCs replace the damaged cells and repopulate in the injured tissues or organs [35]. However, the number of long-term substituted MSCs is open to dispute because those numbers differed depending on the injury models, the transplant route and time, and so on [8]. Nevertheless, it seems to be uncontroversial that MSCs and the CM from MSCs contribute to improving damage from liver injuries [9,36]. The transplanted MSCs help or allow the survival or proliferation of endogenous cells by direct contact or modulating inflammation [9]. MSCs are known to alleviate acute and chronic liver injury by changing the disease environment; for example, they decreased inflammation and cell death, but they increased cell proliferation [9]. In the present study, TSG-6 released by MSCs suppressed the proinflammatory signal and reduced the collagen accumulation which was likely due to the inactivation of hepatic stellate cells by TSG-6 in the acute liver injury of mice.

Although the anti-inflammatory action of TSG-6 is shown to promote improvement in the damaged tissue [14,37], this effect of TSG-6 in the liver remains unknown. Thus, we investigated whether TSG-6 might be involved in liver regeneration, and how it contributed to this process. Our results demonstrated that TSG-containing CM led to liver regeneration. This protective effect of TSG-6 was also observed in another experimental model; specifically, chorionic plate-derived MSCs (CP-MSCs) that shared common characteristics and differentiation potentials with bone marrow-MSCs promoted liver regeneration in rat livers that had been chronically damaged by CCl_4_ [38,39]. In this model, the CP-MSCs contained a greater expression of TSG-6, and the regenerating liver transplanted with CP-MSC showed a higher expression of TSG-6, compared to a non-transplanted liver (Additional file 1: Figure S1). This evidence strongly supported the regenerative effect of TSG-6 in the liver.

In this experimental model, acutely injured livers showed distorted histomorphology, changes in LW/BW, and increased levels of the liver enzymes, ALT and AST, but TSG-6-CM treatment attenuated those abnormalities and even contributed to restoring the liver function which was evidenced by g6pc expression. G6pc hydrolyzes glucose-6-phosphate, which is the central metabolite of glucose metabolism [40]. The data showing almost normal glucose metabolic homeostasis in TSG-6-CM treated livers strongly support the restoration of liver function through the action of TSG-6. In addition, the downregulated proinflammatory factors in the TSG-6-CM group demonstrated that TSG-6 exerted an anti-inflammatory effect on the CCl_4_-treated mouse livers.

Liver injury induces activation of hepatic stellate cells and proliferation of progenitors [16,26]. Activated stellate cells have been shown to be associated with the proliferation of hepatic progenitors [27,41]. In Ki67 staining, more Ki67-positive hepatocytic cells and fewer Ki67–positive hepatic stellate cells were observed in livers of the TSG6 group. Compared with liver of CCl4 + TSG-6-treated mice, livers of CCl4 or CCl4 + NC-treated mice contained more PanCK-expressing cells and more SoX9-expressing cells (Figure 5). We examined if these changes in the activation and /or proliferation of hepatic stellate cells and the reduced liver damage influenced the proliferation of progenitors. As we expected, decreased expansion of progenitors was observed in livers of the TSG-6 group, compared to the CCl4 and NC groups. Therefore, these results indicated that micro-environmental changes by TSG-6 might contribute to the proliferation of hepatocytes by protecting hepatocytes from injury. Also, it could be possible that TSG-6 might promote differentiation of progenitors into hepatocytes. However, neither the direct effects of TSG-6 on progenitors nor the progenitor proliferation at the early time point after CM injection was investigated in the present studies. Hence, further studies are required to provide the evidence for this possibility.

The degree of inflammatory response parallels the fibrotic severity in the liver [42]. The activated hepatic stellate cells play a key role in hepatic fibrogenesis [43,44]. Both RNA and the protein level of fibrotic markers were downregulated in the livers of TSG-6-CM-treated mice. Activated LX2 cultured in TSG-6-CM showed a decreased expression of fibrotic markers and an increased number of lipid drops. Conversely, the addition of the TSG-6 antibody neutralized these inhibitory effects of TSG-6 on the activation of LX2, although this addition was less effective than NC-CM. It is possible that the TSG-6 antibody does not block all TSG-6, and the amount of TSG-6 that escaped blocking by the antibody is higher than it is in NC-CM. One explanation is that the TSG-6-overexpressing MSCs and the CM of those cells show a greater expression of TSG-6, but TSG-6 is rarely detected in NC and NC-CM. Hence, these results proved that TSG-6 influenced the activation of hepatic stellate cells, suggesting that TSG-6 contributed to the reduced fibrosis in damaged livers of mice.

CM obtained from empty plasmid-transfected MSC was less protective in the damaged liver. In line with our finding, Herrera *et al*. [7], demonstrated that both human liver stem cells (HLSCs) and HLSC-CM improved liver injury and had a protective effect for liver, but MSC-CM was totally ineffective in their experimental model [7]. In addition, they employed the concentrated CM in their experimental model of liver injury, like other researchers did. However, the CM used in the present studies was not concentrated. MSC-CM includes many kinds of cytokines, growth factors, and even microparticles [37,45]. Because we used 0.5 ml CM per mouse from a total 12 ml CM which was collected at 70% to 80% confluent cells, this small volume of CM seemed to contain a significantly lower amount of those factors, compared to the concentrated CM. Hence, the effects of other factors were likely negligible in this model. Also, we examined the regulation of hepatic stellate cells by TSG-6, not the apoptosis of hepatocytes, and demonstrated that hepatic stellate cells were inactivated in TSG-6-CM, but not in NC-CM or TSG-6-CM with the TSG-6 antibody. Furthermore, CCl_4_-treated mice injected with TSG-6-CM incubated with TSG-6 antibody induced the liver damage which was ameliorated in CCl_4_-treated mice with TSG-6-CM. These results suggest that the reduced inflammation and fibrosis caused by TSG-6 might provide a beneficial microenvironment which contributes to the proliferation or survival of hepatocytes, leading to liver regeneration.

## Conclusions

Our results first demonstrate that TSG-6 influences liver regeneration and place TSG-6 into the central position in the regulation of liver regeneration. The protective and/or improving effects of TSG-6 on the damaged liver through attenuating inflammation and fibrosis help to develop novel therapeutic approaches to treat acute liver failure. However, further studies are required to examine the underlying mechanism for the regenerative actions of TSG-6 and those effects in other liver diseases, including chronic diseases.

## Additional file

Additional file 1: Figure S1. Chorionic plate-derived MSCs express TSG-6. (A) QRT-PCR analysis for TSG-6 in CP-MSCs (B) QRT-PCR analysis for CP-MSCs-transplanted rat liver which had been chronically damaged by CCl4 (**P* <0.05, ***P* <0.005) (Transplantation: CCl_4_-treated rats with CP-MSCs transplantation).

## Abbreviations

ALT: alanine aminotransferase
ANOVA: analysis of variance
AST: aspartate aminotransferase
CFSE: carboxyfluorescein succinimidyl ester
CM: conditioned medium
FBS: fetal bovine serum
G6pc: glucose-6-phosphatase
H & E: hematoxylin and eosin
hMSC: human mesenchymal stem/stromal cells
IHC: immunohistochemistry
IL: interleukin
NC: negative control
PanCK: pancytokeratin
PBS: phosphage-buffered saline
SD: standard deviation
TGF-β: transforming growth factor-β
TNF-α: tumor necrosis factor-α
TSG-6: tumor necrosis factor-inducible gene 6 protein
α-SMA: α-smooth muscle actin
