# Supplementary material for: Tumor necrosis factor-inducible gene 6 promotes liver regeneration in mice with acute liver injury
Source: Stem Cell Res Ther. 2015 Mar 11;6(1):20. doi: 10.1186/s13287-015-0019-z (PMC4396561; doi:10.1186/s13287-015-0019-z)
Supplement: Additional file 1: Figure S1. — Chorionic plate-derived MSCs express TSG-6. (A) QRT-PCR analysis for TSG-6 in CP-MSCs (B) QRT-PCR analysis for CP-MSCs-transplanted rat liver which had been chronically damaged by CCl4 (*P <0.05, **P <0.005) (Transplantation: CCl4-treated rats with CP-MSCs transplantation). [file 13287_2015_19_MOESM1_ESM.pdf]

Additional file 1

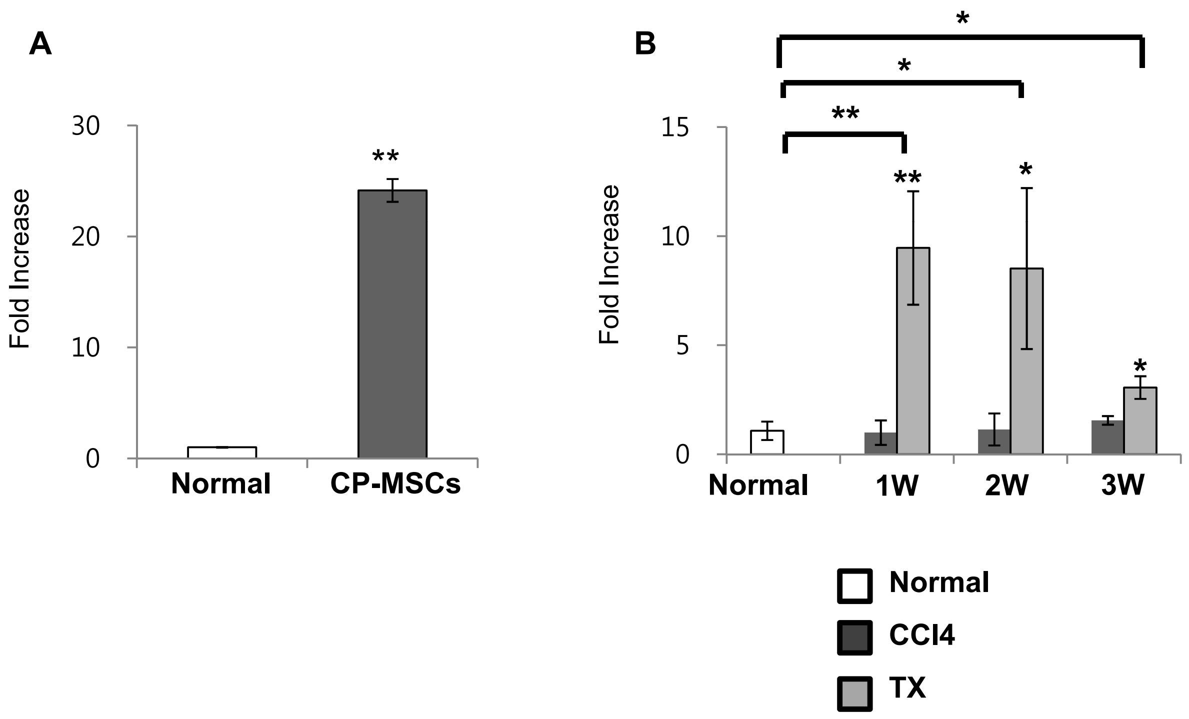

Additional Figure 1. Chorionic plate-derived MSCs expresses TSG-6

(A) QRT-PCR analysis for TSG-6 in CP-MSCs (B) QRT-PCR analysis for CP-MSCs-transplanted rat liver which had been chronically damaged by CCl4 (*p<0.05, **p<0.005) (Transplantation: CCl_4-treated rats with CP-MSCs transplantation).
